# Unsupervised beyond-standard-model event discovery at the LHC with a novel quantum autoencoder

**DOI:** 10.1007/s42484-025-00258-4

**Published:** 2025-03-15

**Authors:** Callum Duffy, Mohammad Hassanshahi, Marcin Jastrzebski, Sarah Malik

**Affiliations:** Physics and Astronomy, University College London, Gower St, London, WC1E 6BT UK

**Keywords:** Quantum autoencoder, High-energy physics, Anomaly detection, Magic, Entanglement entropy

## Abstract

This study explores the potential of unsupervised anomaly detection for identifying physics beyond the standard model that may appear at proton collisions at the Large Hadron Collider. We introduce a novel quantum autoencoder circuit ansatz that is specifically designed for this task and demonstrates superior performance compared to previous approaches. To assess its robustness, we evaluate the quantum autoencoder on various types of new physics ‘signal’ events and varying problem sizes. Additionally, we develop classical autoencoders that outperform previously proposed quantum autoencoders but remain outpaced by the new quantum ansatz, despite its significantly reduced number of trainable parameters. Finally, we investigate the properties of quantum autoencoder circuits, focusing on entanglement and magic. We introduce a novel metric in the context of parameterised quantum circuits, stabiliser 2-Rényi entropy to quantify magic, along with the previously studied Meyer-Wallach measure for entanglement. Intriguingly, both metrics decreased throughout the training process along with the decrease in the loss function. This appears to suggest that models preferentially learn parameters that reduce (but not minimise) these metrics. This study highlights the potential utility of quantum autoencoders in searching for physics beyond the standard model at the Large Hadron Collider and opens exciting avenues for further research into the role of entanglement and magic in quantum machine learning more generally.

## Introduction

A central objective of high-energy physics (HEP) research at the Large Hadron Collider (LHC) is the identification of potential new physics phenomena in the vast datasets generated by collision events. Unsupervised machine learning techniques, trained on standard model (SM) processes, have been utilised to identify whether collision events can be described within the SM framework (Fraser et al. 2022; Ostdiek 2022; Andreassen et al. 2019; Gonski et al. 2022). This focus on unbiased machine learning methods allows us to probe a plethora of new physics scenarios (D’Agnolo and Wulzer 2019; Blance et al. 2019; Mikuni et al. 2022; Govorkova et al. 2022; Hajer et al. 2020). However, with the LHC set to transition into its high-luminosity phase, demand for efficient algorithms capable of processing vast volumes of data is paramount. A potential solution for providing efficient computation may come from the field of quantum computation.

There is compelling motivation for investigating the potential of quantum computing to aid the high-energy physics (HEP) community, with many possible domains of study, such as quantum simulation (Nachman et al. 2021; Bauer et al. 2023) and the analysis of experimental results (Delgado et al. 2022). In this study, we apply quantum machine learning (QML) techniques to the task of identifying anomalous collision events at the LHC, which may signal the presence of physics beyond the standard model.

QML is the integration of machine learning into the framework of quantum mechanics. Considerable promise has emerged from this field. When compared to its classical counterpart, QML can demonstrate comparable, and in some cases superior, performance in specific tasks (Huang et al. 2022). In instances where a performance gap is observed or proven, the term ‘quantum advantage’ has been introduced. Theoretical demonstrations of quantum advantage, particularly from the perspective of computational complexity (Lloyd et al. 2013; Liu et al. 2021), albeit sometimes contrived, have been observed. Furthermore, investigations into sample complexity in generalisation from limited training instances (Caro et al. 2022), along with the exploitation of non-classical correlations within quantum models, a challenge for classical models (Huang et al. 2022; Gao et al. 2022), underscore the anticipated performance advantages of quantum machines.

Despite QML’s promise, there is still much in the field to be resolved, and arguably, one of the largest challenges is trainability. Many of the proposed quantum models suffer from what is known as a barren plateau, where the loss landscape exponentially flattens with model or problem size due to various circuit properties (McClean et al. 2018; Holmes et al. 2022; Cerezo et al. 2021; Thanasilp et al. 2023; Wang et al. 2021). This, in turn, hinders many gradient-based optimisation techniques. Another bottleneck within QML arises due to the infancy of current hardware and the limited number of qubits we can simulate classically.

QML has already been used for various applications to problems in HEP. Supervised learning approaches have been used for event reconstruction (Tüysüz et al. 2020, 2021; Magano et al. 2022; Martínez de Lejarza et al. 2022; Duckett et al. 2024) and classification tasks (Guan et al. 2021; Wu et al. 2021; Terashi et al. 2021; Blance and Spannowsky 2021; Belis et al. 2021) across various problem domains. Similarly, the application of unsupervised learning techniques for detecting beyond-standard model (BSM) signatures has garnered attention (Alvi et al. 2023; Woźniak et al. 2023; Ngairangbam et al. 2022).

Building on these research efforts to detect BSM physics, our work employs a quantum autoencoder (QAE) model. It focuses on identifying a more effective ansatz and exploring larger problem sizes, compared to existing literature, and characterising the inherent properties of these quantum circuits. Previous studies on the QAE successfully identified resonant heavy Higgs signals amidst a QCD $t\bar{t}$ background, leveraging four kinematic features. To expand the scope of QAEs in particle physics, we develop a novel ansatz utilising up to 16 features and also extend the analysis beyond this signal to include gravitons and scalar bosons.

## Autoencoders

The autoencoder was originally introduced as a neural network designed to reconstruct its input from a compressed latent representation (Rumelhart et al. 1986). It compresses data into the lower-dimensional latent representation via a mapping known as the encoder. This compressed representation should capture the input’s essential features, enabling a decoder to regenerate an output that faithfully represents the original input. Crucially, the dimensionality of these latent representations is typically less than that of the input data. This reduction is necessary because it allows the model to distil and encode the underlying structures and patterns within complex, high-dimensional datasets. By focusing on these key attributes of the data, the autoencoder can better generalise from the training data to unseen scenarios during testing (Fefferman et al. 2013). The latent representation produced via the encoder is sometimes called the information bottleneck. We can formally define the learning problem of the autoencoder by the pair of functions *E* and *D*, which satisfy

1 $$\begin{aligned} argmin_{E,D} \mathbb {E}[\Delta (x,D \circ E(x))], \end{aligned}$$

where *E* and *D* denote the encoder and decoder respectively, *x* is the input data and $\Delta$ is the chosen reconstruction loss function.

Many applications exist for autoencoders, including generative modelling and dimensionality reduction, but our primary interest here is anomaly detection (Bank et al. 2020). The general principle of using autoencoders for anomaly detection relies on training the model to accurately reconstruct data originating from some non-anomalous distribution. During testing, the model is exposed to both the distribution seen during training and that from an anomalous new distribution. If the autoencoder was trained successfully, input data from the training distribution should be well reconstructed, while data from the anomalous distribution should be poorly reconstructed. From this discrepancy in reconstruction loss, anomalies should be identifiable.

### Classical

**Fig. 1:**
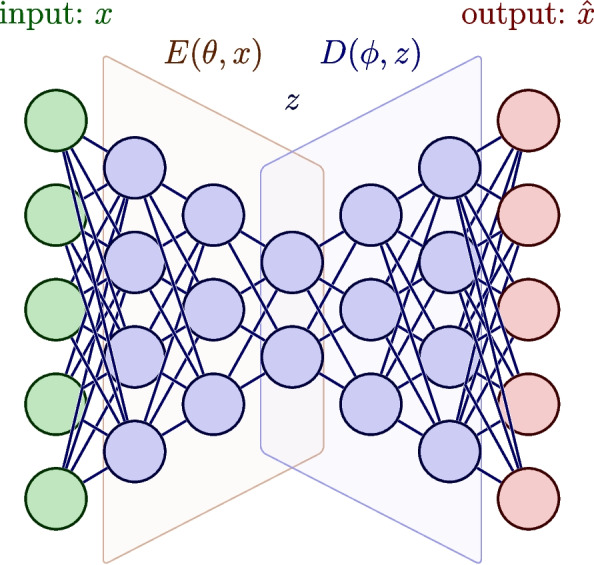
An autoencoder neural network architecture consisting of five input features and a latent space of two. The encoder and decoder each consist of three hidden layers comprising of [4, 3, 2] neurons

Classical autoencoders (CAEs) are a specific type of neural network architecture with two main components: the encoder and the decoder. The architecture of an autoencoder can often be recognised by the decreasing number of neurons in each subsequent hidden layer of the encoder, followed by a mirror image of these layers in the decoder. The encoder, $E(\theta , x)$, receives inputs *x*, which for our purposes are *n*-dimensional vectors. These are then mapped to a compressed latent representation *z*. The resultant latent representation *z* is a vector of dimension *k*, which is strictly less than *n*. The decoder, $D(\phi , z)$, then transforms the latent representation *z* to a vector of dimension *n* in an attempt to reconstruct the input; we denote this output as $\hat{x}$. This is pictorially represented in Fig. 1.

An appropriate loss function must be chosen to train an autoencoder, representing an encoding of the problem solution. For this study, we chose the root mean square error (RMSE)

2 $$\begin{aligned} L(x,\hat{x}) = \sqrt{\frac{\sum _{i=1}^{i=N}(\hat{x}_i - x_i)^2}{N}} \end{aligned}$$

where $x_i$ is an input data point, $\hat{x}_i$ is the corresponding output from the autoencoder, and *N* is the total number of points being considered by the loss function.

Two variants of the CAE were implemented as benchmarks against which to compare the QAE. The first variant is the standard dense neural network, which has varying numbers of hidden layers depending on the problem. The second variant employs a sparse neural network to reduce the number of considered parameters. Due to the all-to-all connectivity between layers of a neural network, the number of parameters grows rapidly with every hidden layer added

3 $$\begin{aligned} N_{params} = \sum _{i=0}^{L-1}l_i l_{i+1} + \sum _{i=1}^{L}l_i, \end{aligned}$$

with *L* being the number of layers and $l_i$ the number of neurons in layer *i*. The first term calculates the number of weights in the network, and the second term counts the number of biases. Given the limitations of current quantum hardware, which cannot support models with as many parameters as the most advanced classical models, we also use sparse networks for a fairer comparison. The sparse neural networks we constructed contained a number of parameters of the same order of magnitude as those in the quantum models. For this purpose, the jaxpruner library was used, which employs magnitude pruning to mask a user-specified percentage of the lowest magnitude weights during training (Lee et al. 2023).

### Quantum

The quantum algorithm we consider belongs to the family of variational quantum algorithms (VQAs), which leverage classical and quantum computing resources. VQAs employ parameterised quantum circuits (PQCs), which are constructed using arbitrary rotation and entangling gates. The parametrised gates of the circuit are optimised. Typically, the PQCs are divided into three parts: a feature map, an ansatz, and a measurement.

Feature maps provide a means for embedding classical data into quantum circuits. Often, input data is encoded as the angle within arbitrary rotation gates. Ideally, when mapping data into a Hilbert space, the mapping should exploit some inherent structure in the data such that data belonging to different classes are maximally separated. However, in this study, our focus was not on the feature maps but on the ansatz design.

The ansatz contains the trainable rotation gates, parametrised by angles $\theta$. By exploiting structural aspects of the data, such as symmetries (Ragone et al. 2023), or considering the form of the loss function and the corresponding roles of qubits within the circuit, we can strategically inform the design of the ansatz. The selection of a specific ansatz influences the inductive bias of the model and, in turn, the range of functions that the model has access to. We denote an ansatz as $U(\theta )$, a unitary that can be further expressed as a series of sequentially applied unitaries

4 $$\begin{aligned} U(\theta )=U_L(\theta _L)U_{L-1}(\theta _{L-1})...U_2(\theta _2)U_1(\theta _1), \end{aligned}$$

where

5 $$\begin{aligned} U_i(\theta _i)=\prod _k e^{-i\theta _k H_k}W_k. \end{aligned}$$

$W_k$ is an unparameterised unitary, $H_k$ is a Hermitian operator, and $\theta _i$ is the $i^{th}$ element in $\theta$.

Measurements must be performed to extract information from a PQC. The outcome of these measurements is then fed into an appropriate cost function, which we denote as $C(\theta )$. We can further express the cost function using the construction above as

6 $$\begin{aligned} C(\theta ) = \sum _k f_k (Tr[O_k U(\theta ) \rho _k U^{\dag }(\theta )]), \end{aligned}$$

where $O_k$ is the measurement operator, $U(\theta )$ is the PQC, $\rho _k$ is the input to $U(\theta )$, and $f_k$ represents the classical post-processing required. Cost functions represent a hypersurface consisting of various minima. Ultimately, the goal is to traverse this hypersurface using a chosen optimiser to converge at the global minimum.

**Fig. 2:**
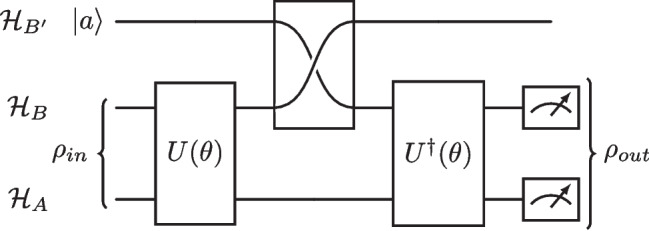
A general schematic of the quantum autoencoder. Input states, $\rho _{in}$, are derived from a feature map and then processed by the unitary ansatze $U(\theta )$, $U^\dag (\theta )$. The Hilbert spaces $\mathcal {H}_A$, $\mathcal {H}_B$ and $\mathcal {H}_{B'}$ are the latent space, the trash space, and the reference space, respectively

The quantum autoencoder (QAE) is a form of VQA and was conceptualised as the quantum analogue of the CAE (Romero et al. 2017). Both share a similar feature: the compression of information. In the case of the QAE, this involves compressing quantum information into a smaller Hilbert space while preserving features of the greatest importance. As a learning task, the final reconstructed state $\rho _{out}$ should faithfully represent the initial input state $\rho _{in}$. This accuracy can be checked by calculating the fidelity $F(\rho _{in}, \rho _{out})$.

A schematic of a QAE can be found in Fig. 2. The model can be described in terms of three subspaces: $\mathcal {H}_A$ is the latent space, $\mathcal {H}_B$ is sometimes referred to as the ‘trash’ space and represents the space that will be discarded, and $\mathcal {H}_{B'}$ is a reference space required to erase information in $\mathcal {H}_B$. The role of $\mathcal {H}_{B'}$ is described in detail later in this section. The encoder component of this algorithm is described by the circuit $U(\theta )$, which takes trainable parameters as arguments and acts across $\mathcal {H}_A \otimes \mathcal {H}_B$. To achieve data compression, the set of qubits in $\mathcal {H}_B$ is disregarded after the action of $U(\theta )$. One should note here that disregarding $\mathcal {H}_B$ is not done in practise, the reasons for which will be a detailed layer. Once we have performed the compression, the next step is to decode the latent representation to the original dimension of the input state, a task performed by a decoder $U^{\dag }(\theta )$. Since $U^{\dag }(\theta )$ acts on the space $\mathcal {H}_A \otimes \mathcal {H}_B$ and the objective of the decoder is to extract useful information solely from the compressed space $\mathcal {H}_A$, the information contained in $\mathcal {H}_B$ must be erased. To achieve this, the information in $\mathcal {H_B}$ is swapped with that in $\mathcal {H_{B'}}$, which contains some reference state $|a\rangle$. This is analogous to erasing the information in $\mathcal {H}_B$. Once this is done and the decoder $U^{\dag }(\theta )$ is applied, we retrieve a state $\rho _{out}$, which, after the parameters $\theta$ have been optimised, should faithfully represent the input state we began with.

To formalise the above process into a cost function that needs to be minimised, we use

7 $$\begin{aligned} C_1(\theta ) = \frac{1}{N}\sum _{i=1}^N(1-F(\rho _{i, in}, \rho _{i, out})), \end{aligned}$$

where $F(\rho _{i, in}, \rho _{i, out})$ is the fidelity between the input $\rho _{i, in}$ and output $\rho _{i, out}$ states, and the input data is encoded into input states $\rho _{i, in}$.

It can be shown that the quantum autoencoder architecture can be simplified to a form which does not make use of $U^{\dag }(\theta )$ explicitly (Ngairangbam et al. 2022; Romero et al. 2017). A simple explanation of this method begins with identifying an encoder unitary which performs perfect compression. Specifically, for all input states, it produces the following:

8 $$\begin{aligned} U(\theta )|\psi _i\rangle _{AB} = |\psi _i^c\rangle _A \otimes |a\rangle _B, \end{aligned}$$

where $|\psi _i^c\rangle _A$ is the compressed state, and $|a\rangle _B$ is the reference state. Upon the action of $U^{\dag }(\theta )$, $|\psi _i\rangle _{AB}$ will be recovered for all *i* since the unitary $U(\theta )$ has been able to produce the reference state $|a\rangle$ in $\mathcal {H}_B$ for all input states. As a result, for the learning task, it suffices to consider whether, after performing $U(\theta )$ the resultant state in $\mathcal {H}_B$ matches that of the reference state in $\mathcal {H}_{B'}$. To achieve this, we compute the fidelity between the two states. From the above, we can re-state the cost function as

9 $$\begin{aligned} C_2(\theta ) = \frac{1}{N}\sum _{i=1}^N (1 - F({{\,\textrm{Tr}\,}}_A[U(\theta )\rho _{i,in}U^{\dag }(\theta )], |a\rangle \langle a|_{B'})). \end{aligned}$$

**Fig. 3:**
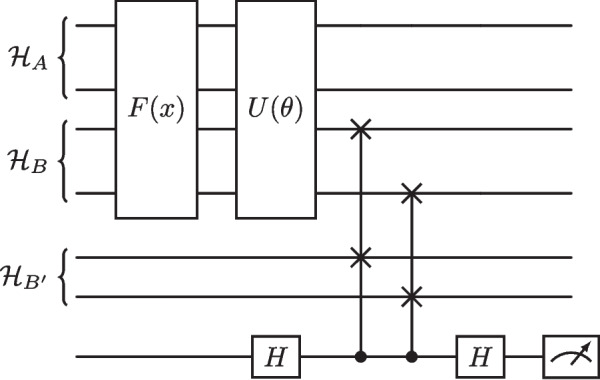
A diagram showing the form of the autoencoder used in practise. Here, we have four input qubits and a latent space of two qubits. Initial input states $|\psi _i\rangle$ are formed from a feature map *F*(*x*) embedding classical data in a Hilbert space. States created by *F*(*x*) are then passed into the chosen ansatz $U(\theta )$. Finally, a SWAP test is performed between the spaces $\mathcal {H}_B$ and $\mathcal {H}_{B'}$ to extract information from the circuit

For implementing the fidelity measurement in practice between $\mathcal {H}_B$ and $\mathcal {H}_{B'}$, the swap test is used and is shown in Fig. 3 after the action of a feature map *F*(*x*) and ansatz $U(\theta )$. The reference state is chosen to be the all-zero state.

**Fig. 4:**
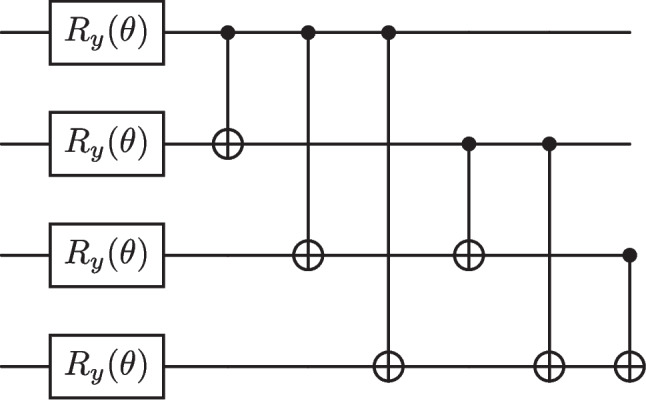
The all-to-all entangling ansatz, which contains parameterised Pauli-Y rotation gates followed by CNOT gates that entangle all pairs of qubits to one another

For this paper, we propose an ansatz well suited to the task of a QAE. To demonstrate its effectiveness, we compare it to two popular ansatz. In all parameterised circuits used, the rotation gates are kept the same, but the entangling structure differs.

**Fig. 5:**
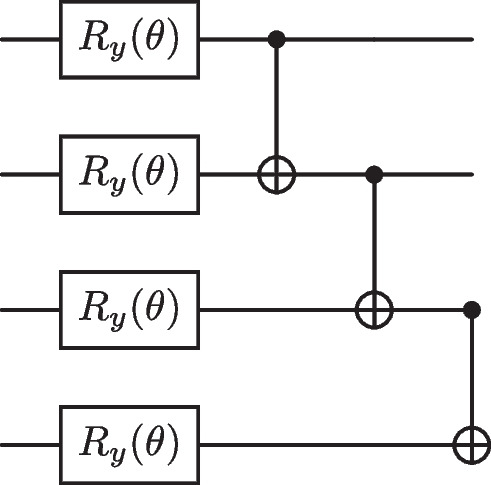
The HEA, which contains parameterised Pauli-Y rotation gates followed by CNOT gates that entangle neighbouring pairs of qubits to respect the qubit connectivity of real quantum devices

The first of these ansatz can be seen in Fig. 4, depicting all-to-all entangling CNOT gates in the case of four qubits, to spread correlations amongst all qubits. The number of CNOT gates required scales quadratically with the number of qubits. This circuit was considered in previous work when dealing with anomaly detection in particle physics (Ngairangbam et al. 2022), and we will refer to this ansatz from here on as the original ansatz. The second ansatz, the hardware efficient ansatz (HEA), contains CNOT gates between nearest neighbour qubits. A four-qubit example of this ansatz can be seen in Fig. 5. The idea behind this structure is to respect the connectivity of many near-term quantum devices to minimise the effect of hardware noise (Leone et al. 2024).

**Fig. 6:**
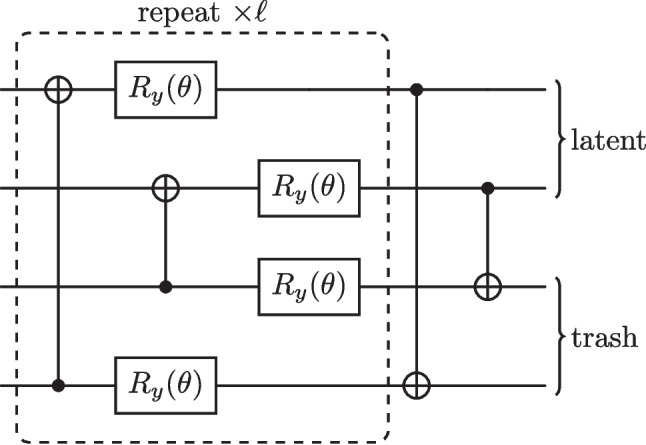
The newly proposed ansatz. Initially, CNOT gates link every qubit in the trash space to those in the latent space, followed by parameterised arbitrary Pauli-Y rotation gates. From here, a second set of CNOT gates which act on every qubit in the latent space with target bits lying in the trash space. This ansatz is aware of which qubits belong to $\mathcal {H}_A$ and which to $\mathcal {H}_B$, adapting where CNOT gates act accordingly

The ansatz we propose contains CNOT gates both before and after the rotation gates, as shown in Fig. 6, for the specific case of four qubits, with a latent and trash space of two qubits. Recent literature has stressed the importance of inductive biases in quantum machine learning models, whereby restricting the class of functions the model has access to can improve generalisation performance (Ragone et al. 2023). The choice of a unitary ansatz directly influences the class of functions the model can produce. Here, the structure of the circuit is informed by the role of each qubit, such as whether it belongs to the latent space or if its information will be discarded. The initial set of CNOT gates aims to transfer information contained in qubits that belong to $\mathcal {H}_B$ to qubits that will remain in the latent space $\mathcal {H}_A$. Here, we have chosen the control bit to lie in $\mathcal {H}_B$ and the target bit in $\mathcal {H}_A$. This choice only becomes relevant for the special case of the input state being perfectly compressed into $\mathcal {H}_A$, leaving $\mathcal {H}_B$ in the all-zero state. Subsequent CNOT gates will then no longer alter the state.

Both sets of entangling gates (before and after rotations) have been found to be crucial for the enhanced performance of the ansatz. If one aims to have $\ell$ layers of $R_y(\theta )$ gates, the CNOT gates performing information transfer are repeated $\ell$ times, with the final layer also containing CNOT gates which erase information in the qubits to be discarded. This is a step towards constructing a more standardised ansatz for QAEs, which has been highlighted as an important goal in the literature (Caro et al. 2022). We focus on producing an ansatz with fewer CNOT gates compared to the all-to-all ansatz and select those considered most useful for the computation at hand. This approach prioritises the transfer of information between the latent and trash spaces, thereby biasing the model to compress the most valuable information into the latent space in as few layers as possible. This design is not only advantageous from a hardware perspective but may also help delay the onset of entanglement-induced barren plateaus. In the original ansatz, the number of layers required to approximate a 2-design scales logarithmically with system size (Boixo et al. 2018), eventually leading to entanglement-induced barren plateaus due to the high qubit connectivity. Seeing whether this new ansatz can achieve deeper circuits before encountering this form of barren plateau could be an interesting direction of future study but is beyond the scope of this work.

Further details on how this ansatz should be implemented for a chosen latent space size, number of layers, and qubits are in Appendix A. Though the focus of this paper pertains to anomaly detection at the LHC, the ansatz we have designed here is general and could be applied to other domains since it does not account for potential structure in data at the LHC.

## Circuit properties

In addition to analysing the anomaly detection performance of the circuits discussed, two information-theoretic properties of interest are investigated. These are as follows: entanglement and magic. Both entanglement and magic provide the means to quantify the amount of quantum resources we are utilising, which in turn, can be used as indicators of potentially unwanted behaviour of trained circuits. While it is currently not possible to conclusively determine that a specific quantum circuit cannot be simulated classically simply by calculating these metrics, these metrics can still provide useful insights. Obtaining maximal entanglement or magic can be an indication of undesirable circuits. In the case of entanglement, it has been shown that circuits which possess volume-law entanglement scaling lead to barren plateaus (Ortiz Marrero et al. 2021). As for magic, it has been shown Haar random states approach maximal magic (Liu and Winter 2022). Thus, a circuit producing mostly high-magic states could be suspected to be Haar random and therefore undesirable (McClean et al. 2018).

### Entanglement

Generating entanglement is a necessary but not sufficient condition for quantum circuits that aim to achieve a quantum advantage. In quantum machine learning, entanglement is often employed to capture non-trivial correlations in data (Schuld et al. 2020; Kandala et al. 2017). CNOT and CZ gates are commonly used to realise entanglement in quantum circuits.

To empirically measure entanglement, the Meyer-Wallach entanglement measure (Meyer and Wallach 2002) can be used. This is a global measure of multi-particle entanglement for pure states, aggregating bipartite entanglement measures across different subsystems. Often denoted as *Q*, the Meyer-Wallach entanglement measure can be defined as follows: Consider an *n* qubit system with a linear map $l_j(b)$ acting on the computational basis states

10 $$\begin{aligned} l_j(b)|b_1 ... b_n\rangle = \delta _{bb_j}|b_1...\hat{b}_j ... b_n \rangle , \end{aligned}$$

where $b_j \in \{0,1\}$ and $\hat{\phantom{0}}$ indicates qubit j being absent. The entanglement measure can then be defined as

11 $$\begin{aligned} Q(|\psi \rangle ) = \frac{4}{n}\sum _{j=1}^{n}D(l_j(0)|\psi \rangle ,l_j(1)|\psi \rangle ), \end{aligned}$$

with

12 $$\begin{aligned} D(|u\rangle ,|v\rangle ) = \frac{1}{2}\sum |u_i v_j - u_jv_i|^2, \end{aligned}$$

$|u\rangle =\sum u_i |i\rangle$ and $|v\rangle = \sum v_i|i\rangle$. *D* can be seen as a distance operator. The measure defined here has the following properties:

- Invariant under local unitaries (acts independently on each subspace, thus composed of only single-qubit unitaries).
- $$0 \le Q \le 1$$.
- $Q(|\psi \rangle ) = 0$ iff $|\psi \rangle$ is a product state.
- $Q(|\psi \rangle )$ approaches 1 for highly entangled states $|\psi \rangle$. For example the GHZ state has $Q(\frac{|0\rangle + |1\rangle }{\sqrt{2}} = 1)$.

This measure can be shown to be equivalent to $Q(|\psi \rangle ) = 2(1 - 1/n \sum _{k=0}^{n-1}Tr[\rho _k^2])$, which is an average over entanglements of each qubit with the rest of the system, for a system of *n* qubits (Brennen 2003). Previous work has used this measure to characterise entanglement in error correcting codes and quantum phase transitions (Meyer and Wallach 2002; Somma et al. 2004). This definition has been extended to characterise parametrised quantum circuits by taking the average of the Meyer-Wallach entanglement measure over a collection of sampled parameters, more formally (Sim et al. 2019)

13 $$\begin{aligned} \bar{Q}(|\psi (\theta )\rangle ) = \frac{1}{|S|}\sum _{\theta \in S}Q(|\psi (\theta )\rangle ), \end{aligned}$$

where $S\in \{\theta \}$ is the set of sampled circuit parameters. Here, only the ansatz of the quantum circuit is considered.

For this study, we consider the circuit as a whole, including both the ansatz and feature map. Focusing solely on the ansatz neglects that it acts on product or entangled states generated via the feature map. As a result, we study the distribution of global entanglement as a function of both parameters $\theta$ and data *x*, $Q(|\psi (\theta , x)\rangle )$. From here, there are two distributions of interest to us, the first where we fix $\theta$ to be the set of parameters found after training, giving rise to a distribution over $Q(|\psi (x)_{\theta _{trained}}\rangle )$. This will give us insight into the global entanglement distribution for an optimised circuit over a set of sampled data points. We will also define the sample mean of this distribution as

14 $$\begin{aligned} \bar{Q}(|\psi (x)|_{\theta _{trained}})\rangle ) = \frac{1}{|D|}\sum _{x \in D}Q(|\psi (x)|_{\theta _{trained}}\rangle ), \end{aligned}$$

where $D\in \{x\}$ is the set of sampled data points.

The second distribution we will define is that over both *x* and $\theta$, $Q(|\psi (x,\theta )\rangle )$. We also define the sample mean global entanglement of this distribution by

15 $$\begin{aligned} \bar{Q}(|\psi (x, \theta )\rangle ) = \frac{1}{|S||D|}\sum _{\theta \in S}\sum _{x\in D}Q(|\psi (\theta , x)\rangle ), \end{aligned}$$

where $\theta$ is sampled from the uniform distribution. By comparing Eqs. 14 and 15, we will be able to ascertain whether circuits with optimised parameters preferentially select states with high or low global entanglement.

### Magic

Like entanglement, the magic a quantum circuit possesses is a necessary but not sufficient condition to avoid classical simulatability. The notion of magic arises from the fact that by the Gottesman-Knill theorem, circuits consisting of gates from the Clifford group can be simulated efficiently classically even if they are created with high levels of entanglement (Gottesman 1998). Therefore, creating circuits that are not efficiently simulatable requires the use of non-Clifford gates. These non-Clifford gates can come in the form of *T* and Toffoli gates. Implementing these resources gives quantum computers the ability to outperform classical machines, which has been dubbed as providing the circuit with ‘magic’. Subsequently, a resource theory for magic has been formulated (Leone et al. 2022; Campbell 2011). We can define a measure of magic via the stabiliser 2-Rényi entropy

16 $$\begin{aligned} M_2 (|\psi \rangle ) := - \log _2 W(|\psi \rangle ) - S_2(|\psi \rangle ) - \log _2(d), \end{aligned}$$

where $W(|\psi \rangle ):= {{\,\textrm{Tr}\,}}(Q|\psi \rangle ^{\otimes 4})$, $Q:= d^{-2}\sum _P P^{\otimes 4}$ and $d=2^n$, the sum is taken over all multi-qubit strings of the Pauli operators to four copies of the state and $S_2(|\psi \rangle ) = -\log _2({{\,\textrm{Tr}\,}}(|\psi \rangle ^2))$ is the 2-Rényi entropy. This measure was originally proposed in ref. Leone et al. (2022), along with an experimental protocol; later, this protocol was improved by ref. Oliviero et al. (2022) to only include randomised single-qubit measurements rather than global multi-qubit measurements and then further by Haug et al. (2024). The measure has been proven to be a resource monotone (an accurate measure of non-stabilisesness) (Leone and Bittel 2024). For this work, we calculated the analytic value of magic via Eq. 16 with state vector simulation and did not use the experimental protocols.

This measure of magic has the following properties:

- $M_2(|\psi \rangle )=0$, in the case all the gates in the circuit come from the Clifford group.
- $M_2(C|\psi \rangle )=M_2(|\psi \rangle )$, invariant under Clifford rotations.
- $$0\le M_2(|\psi \rangle ) \le \log (d+1) -\log (2)$$.

In the same spirit as global entanglement measures, we propose to take averages over a set of parameters and data points to gain intuition about the magic generated by our quantum circuits. We construct similar expressions for the magic measure

17 $$\begin{aligned} \bar{M}_2(|\psi (x)|_{\theta _{trained}}\rangle ) = \frac{1}{|D|}\sum _{x\in D}M_2(|\psi (x)|_{\theta _{trained}}\rangle ). \end{aligned}$$

and

18 $$\begin{aligned} \bar{M}_2(|\psi (x,\theta )\rangle ) = \frac{1}{|S||D|}\sum _{\theta \in S}\sum _{x\in D}M_2(|\psi (\theta , x)\rangle ). \end{aligned}$$

## Datasets

The datasets under consideration consist of kinematic information describing proton-proton particle collisions. The two main processes being examined are events well characterised by QCD and those from beyond standard model (BSM) processes. Events originating from BSM processes cannot be well described within the theory of QCD and, for this study, are the signals of interest. Meanwhile, events described by QCD theory will be designated as the in-distribution background data.

The form of the data we are considering is kinematic data from particle collisions, thus classical. However, it is important to realise the classical data used here originates from quantum processes.

By considering two separate datasets and feature maps, we hope to demonstrate the performance advantages of the newly proposed ansatz design, which could be broadly applicable, at least in the context of HEP data. We will now introduce the two datasets we studied, which consist of four signals in total.

### Heavy Higgs

This dataset^1^ is taken from Ngairangbam et al. (2022), which considered the background QCD process of $t\bar{t}$ production $pp\rightarrow t\bar{t}$, with signals originating from scalar resonance production $pp \rightarrow H \rightarrow t\bar{t}$. The signal of interest here involves a heavy Higgs of mass $m_H = 2.5$Tev. All top quarks decay to a bottom quark and a W boson, which decays to a muon. Both events were generated according to a centre of mass energy of 14Tev via MadGraph5 aMC@NLO (Alwall et al. 2014). Showering and hadronisation were taken care of by Pythia8 (Sjöstrand et al. 2015), while Delphes3 was utilised for detector simulation (de Favereau et al. 2014).

Object reconstruction was implemented with the anti-$k_t$ algorithm with a jet radius *R*=0.5. Bottom jets originating from signal events were required to have $p_T^b > 30$Gev with isolation criteria of $R=0.5$. The variables extracted from this process were as follows:

- Two lepton transverse momenta $p_T^{l_1}$, $p_T^{l_2}$.
- Angle between two leptons $\theta _{l}$.
- Two quark transverse momenta $p_T^{q_1}$, $p_T^{q_2}$.
- Angle between two quarks $\theta _{q}$.
- Transverse energy $$E_T$$.
- Two lepton angular separations $dR_{l_1}$, $dR_{l_2}$.

**Fig. 7:**
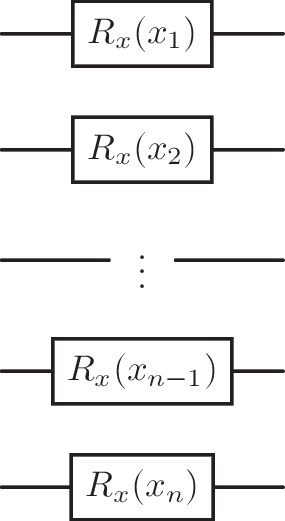
A feature map for the heavy Higgs dataset consisting solely of $R_x$ rotation gates to embed features *x*

Each variable was mapped to the interval $[0, \pi ]$ for QAEs and [0, 1] for CAEs, where non-angular variables are fixed between [0, 1000] by adding two artificial extreme data points before scaling. The faux data is then removed. A reasonably simple feature map was implemented to embed the data from this dataset, consisting solely of $R_x(\theta )$ rotation gates seen in Fig. 7.

The previous study, which utilised this dataset as well as the QAE, used a subset consisting of four kinematic variables $\{E_T, p_T^{b_1}, p_T^{l_1}, p_T^{l_2}\}$. For our study, we studied three subsets of increasing size:

- $$\{E_T, \theta _l, p_T^{l_1}, p_T^{l_2}\}$$,
- $$\{E_T, p_T^{b_1}, p_T^{l_1}, p_T^{l_2}, \theta _l, p_T^{b_2}\}$$,
- $$\{E_T, p_T^{b_1}, p_T^{l_1}, p_T^{l_2}, \theta _l, p_T^{b_2}, \theta _b, dR_{l_1}\}$$,

with the largest subset containing eight features.

### Randall-Sundrum gravitons and ZZZ scalar bosons

A previous study into anomaly detection with quantum kernels created the second dataset^2^ we considered (Belis et al. 2023; Woźniak et al. 2023). The study was yet again into proton-proton collisions at a centre of mass energy of 13Tev, but in this case, the BSM processes were as follows:

- Narrow Randall-Sundrum graviton decaying to two *W*-bosons, Narrow $G \rightarrow WW$ (Randall and Sundrum 1999). The width of the graviton was set to a negligible value by fixing $\kappa m_G$ to 0.01, where $\kappa$ is a dimensionless coupling parameter (Bijnens et al. 2001).
- Broad Randall-Sundrum graviton decaying to two W-bosons, Broad $G \rightarrow WW$ (Randall and Sundrum 1999). The parameter $\kappa m_G$ was set to 2.5, resulting in a width-to-mean ratio of $~35\%$ for $m_{jj}$ after detector effects.
- Scalar boson *A* decaying to a Higgs and a *Z* boson, $A \rightarrow HZ \rightarrow ZZZ$.

All *W* and *Z* bosons are set to decay to quark pairs, producing all-jet final states. The background processes simulated were QCD multi-jet production.

The Delphes3 library was used to simulate detector effects mimicking the CMS detector description, and a proton in-time pileup of 40. The size of the dataset produced equates to an integrated luminosity of $64\text {fb}^{-1}$.

A particle-flow algorithm followed by the anti-$k_t$ clustering algorithm (R=0.8) was employed to process the events. To make the dataset representative of LHC event selection, only dijet events satisfying the invariant mass condition $m_{jj} > 1260$GeV are selected. Moreover, dijets must satisfy $p_T > 200$GeV and $|\nu |<2.4$. Events comprise the two highest $p_T$ jets, with each jet further comprising the 100 highest $p_T$ particles zero-padded when necessary. The kinematic variables used to describe each particle within a jet are $p_T$, $\eta$, and $\phi$.

Since the dataset described above results in a single jet represented by a $100\times 3$ matrix, dimensionality reduction is required to allow current NISQ devices to process the data (Preskill 2018). The method implemented by ref. Woźniak et al. (2023) was a 1D convolutional autoencoder, to respect the structure of the jet data. Jet objects from the QCD background data in the sideband region are compressed, resulting in a reduced dijet dataset of 2*n* features, with *n* being the dimension of the latent representation of each jet. They argue that dimensionality reduction of this nature can partially retain non-linear correlations in the latent space compared to the likes of other dimensionality reduction methods such as PCA.

**Fig. 8:**
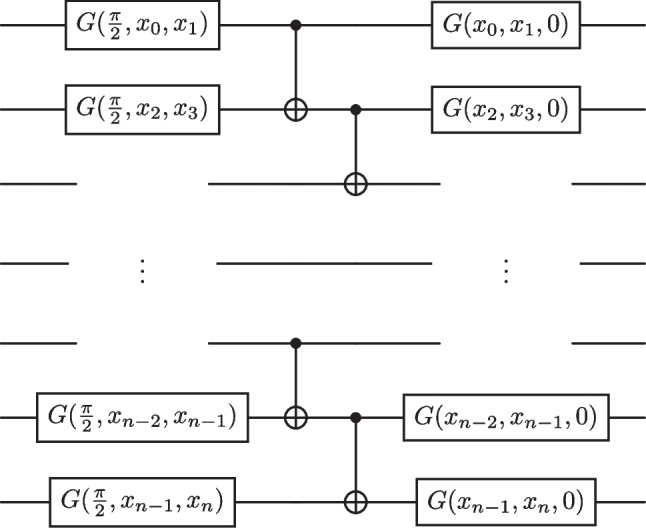
A feature map for the graviton and scalar boson dataset consisting solely $G(\phi , \theta , \omega )$ rotation gates ($G(\phi , \theta , \omega ) = R_z(\omega ) R_y(\theta ) R_z(\phi )$) and nearest neighbour entangling gates

The feature map used, as seen in Fig. 8, is the same as in the original study to focus on the effects of the newly proposed ansatz.

## Results and discussion

The following QAE models presented were created using Pennylane (Bergholm et al. 2022), while the CAEs were built using the Jax ecosystem, utilising Flax and Optax (Bradbury et al. 2018; Heek et al. 2023; DeepMind et al. 2020). For training, 1000 background samples were used, and for testing, 10, 000 samples were used equally split between signal and background sources.

**Fig. 9:**
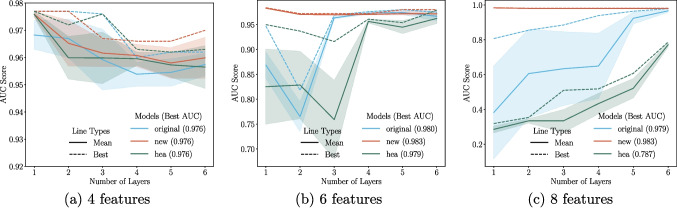
Each subplot depicts the AUC score of a QAE with a particular ansatz as a function of the number of layers, reporting the mean (solid line), standard deviation on the mean (shaded region), and the best AUC score (dashed line), for a set of input features. For each ansatz at each layer, the model was run for 10 different random seeds. All ansatz made use of the same feature map in Fig. 7. **a** Four input features with kinematic variables $\{E_T, p^{b_1}_{T}, p^{l_1}_{T}, p^{l_1}_{T}\}$. **b** Six input features with kinematic variables $\{E_T, p^{b_1}_{T}, p^{l_1}_{T}, p^{l_1}_{T}, \theta _l, p^{b_1}_{T}\}$. **c** Eight input features with kinematic variables $\{E_T, p^{b_1}_{T}, p^{l_1}_{T}, p^{l_1}_{T}, \theta _l, p^{b_1}_{T}, \theta _b, dR_{l1}\}$

The classical models presented have been found from a randomised grid search at each input feature size. Shallow models were found from the best-performing models containing equal to or fewer than 100 parameters. Deep models could exceed the limit of 100 parameters but were restricted to fewer than 1000 parameters so as not to enter the overparameterised regime. Overparameterisation can, in certain instances, enhance neural networks by increasing their capacity to memorise and interpolate data. However, since we do not include overparameterised QAEs in this study, incorporating overparameterised neural networks would shift the focus of the comparison from differences in computational paradigms to disparities in model capacity. The quantum models were also found from the result of a randomised grid search; we refer the reader to Appendix B for details on both the quantum and classical models found from the grid searches. The nature of the grid searches can be found in Appendix C. All models were trained for 500 epochs with the Adam optimiser.

### Performance

#### Heavy Higgs

We begin by evaluating the performance of QAEs in distinguishing the heavy Higgs signal from the QCD background. To compare the efficacy of the original ansatz, the HEA, and the newly proposed ansatz, we plot the AUC score as a function of the number of layers, ranging from one to six, for input feature sizes of four, six, and eight. Each plot, shown in Fig. 9, reports the mean AUC (solid line), standard deviation (indicated by shaded regions), and the best-performing model (dashed lines) for each ansatz, calculated from ten random seeds.

**Table 1:** AUC scores for identifying the heavy Higgs signal for a collection of quantum and classical autoencoders on a test data for a given set of input features

Model	4 features (# params)	6 features (# params)	8 features (# params)
New QAE	0.976 (4)	0.983 (6)	0.983 (8)
Shallow CAE	0.941 (59)	0.968 (70)	0.962 (76)
Deep CAE	0.971 (135)	0.971 (540)	0.976 (942)

The first column considers four input features with kinematic variables $\{E_T, p^{b_1}_{T}, p^{l_1}_{T}, p^{l_1}_{T}\}$. The second column considers six input features with kinematic variables $\{E_T, p^{b_1}_{T}, p^{l_1}_{T}, p^{l_1}_{T}, \theta _l, p^{b_1}_{T}\}$. The third column considers eight input features with kinematic variables $\{E_T, p^{b_1}_{T}, p^{l_1}_{T}, p^{l_1}_{T}, \theta _l, p^{b_1}_{T}, \theta _b, dR_{l1}\}$. We also indicate the number of parameters contained in each model in brackets next to the AUC score

For the case of four input features encoded onto four qubits, all three QAEs achieve their highest AUC of 0.976 with just a single layer, performing similarly thereafter. However, as the input features and corresponding qubit count increase, the performance trends begin to diverge. When six features were used, the newly proposed ansatz consistently outperformed the original and HEA ansatzes, achieving a peak AUC of 0.983. Notably, the original ansatz required three layers to reach a competitive performance, while the HEA only achieves its best performance of 0.979 after six layers.

At eight input features, the performance gap widens further. The proposed ansatz maintains its peak AUC of 0.983, demonstrating both high performance and stability across different seeds. In contrast, the original ansatz reaches its maximum AUC of 0.980 after six layers, while the HEA achieved a maximum AUC of 0.787 after six layers. This under-performance of the HEA highlights its limitations in effectively encoding higher-dimensional input data.

In addition to achieving higher AUC scores, the new ansatz exhibits significantly lower variation across runs, as reflected by its narrower standard deviation bands.

Scores below 0.5, observed at certain layer depths for the original and HEA ansatzes, indicate performance worse than random guessing. This can be attributed to the models’ inability to effectively learn the background data during training, which may result from suboptimal solution spaces in these ansatz designs. Despite having identical trainable rotation gates, the differences in entangling structures among the ansatzes critically impact their ability to represent the underlying data distribution. These results underscore the importance of the entangling structure in determining the efficacy of QAEs in this context.

We then considered the performance of both CAEs and the new QAE to identify the heavy Higgs signal from the QCD background. The number of input features considered was four, six, and eight, with the resulting AUC scores in Table 1. As illustrated in Table 1, the newly introduced QAE ansatz outperformed all classical models at each input size. At four input features, this distinction is particularly evident when considering the shallow network but less so for the deep model. For models with six and eight features, the performance gap narrows between the CAEs and the new QAE but is still evident.

Finally, we can see that the number of parameters contained in the QAEs is far fewer than in the CAEs, even as the problem size increases. This interesting point will be discussed in more detail in the context of the second data set.

**Fig. 10:**
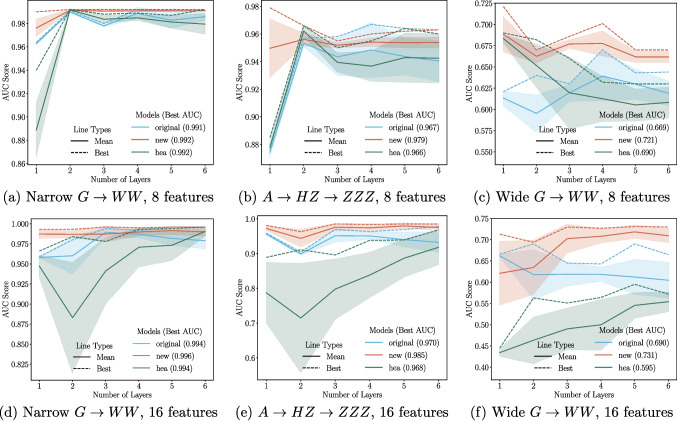
Each subplot depicts the AUC score of a QAE with a particular ansatz as a function of the number of layers, reporting the mean (solid line), standard deviation on the mean (shaded region), and the best AUC score (dashed line), for a set of input features and BSM signals. Each column corresponds to identifying a different BSM signal; going from left to right, we have the narrow graviton, scalar *ZZZ* boson, and wide graviton respectively. The QAEs depicted here each contain the same feature map seen in Fig. 8 but differing ansatz design. For each ansatz at each layer, the model was run for 10 different random seeds. Panels a, b, and c depict models with eight input features. Panels d, e, and f depict models with 16 input features

**Table 2:** A table of the AUC score achieved for the new QAE and CAEs at 8 input features encoded onto 4 qubits for various BSM signals

Model (#params)	Narrow $G \rightarrow WW$	$A \rightarrow HZ \rightarrow ZZZ$	Wide $G\rightarrow WW$
New QAE (4)	0.992	0.979	0.721
Shallow CAE (15)	0.990	0.933	0.529
Deep CAE (183)	0.991	0.939	0.535

Each column corresponds to identifying a different BSM signal; going from left to right, we have the narrow graviton, scalar *ZZZ* boson, and wide graviton respectively. The QAE here contains the same feature map seen in Fig. 8 and ansatz design from Fig. 6. The CAEs come in two flavours shallow and deep some of which have been sparsified to reduce the number of parameters

#### Gravitons and scalar bosons

Following the analysis of the heavy Higgs signal, we evaluate the performance of the introduced ansatz in identifying graviton and scalar boson signals. Figure 10 presents the AUC scores of the quantum models as a function of the number of ansatz layers as we did previously in Fig. 9, highlighting the distinction between the new and older ansatz designs.

We first examine the case of eight input features encoded onto four qubits. For identifying the narrow graviton, the new ansatz achieves its peak performance after a single layer, while the original and HEA ansatzes require two layers to match this performance. A similar trend is observed for identifying the scalar boson: the new ansatz achieves its best performance (AUC = 0.979) with a single layer, whereas the other ansatzes require two layers to reach AUC scores that, within the standard deviation, coincide with the performance of the new ansatz. Notably, neither the original nor the HEA ansatz surpasses the performance of the new ansatz with a single layer. For the wide graviton, the new ansatz consistently maintains its performance as the number of layers increases, while the HEA ansatz matches this performance only up to two layers. The original ansatz, however, fails to achieve comparable AUC scores across the range of layers considered.

**Table 3:** A table of the AUC score achieved for the new QAE and CAEs at 16 input features encoded onto 8 qubits for various BSM signals

Model (#params)	Narrow $G \rightarrow WW$	$A \rightarrow HZ \rightarrow ZZZ$	Narrow $G \rightarrow WW$
New QAE (24)	0.996	0.985	0.731
Shallow CAE (42)	0.997	0.978	0.597
Deep CAE (537)	0.997	0.978	0.641

Each column corresponds to identifying a different BSM signal; going from left to right, we have the narrow graviton, scalar *ZZZ* boson, and wide graviton respectively. The QAE here contains the same feature map seen in Fig. 8 and ansatz design from Fig. 6. The CAEs come in two flavours shallow and deep some of which have been sparsified to reduce the number of parameters

When the number of input features is increased to 16, requiring eight qubits, the performance trends diverge further. For the narrow graviton, the original ansatz requires three layers to match the performance of the new ansatz, while the HEA ansatz takes four layers. In identifying the scalar boson, the original ansatz requires three layers to coincide with the new ansatz, whereas the HEA ansatz requires six. Both older ansatz designs struggle significantly with the wide graviton case, showing minimal performance improvement as the number of layers increases. By contrast, the new ansatz achieves a maximum AUC score of 0.731 after three layers and maintains this performance as additional layers are added.

Across both datasets, we consistently observe that the new ansatz achieves comparable or superior performance with fewer layers compared to the original and HEA ansatzes. This underscores the advantage of the new ansatz in enabling more compact and efficient QAE designs.

For the HEA ansatz, this trend can be understood through a lightcone argument: because the HEA only includes nearest-neighbour entanglement, multiple layers are required to propagate information from qubits in the trash space to those in the latent space. Until this transfer is complete, the QAE cannot fully utilise the information contained in the input features. In contrast, the new ansatz facilitates immediate information transfer between the trash and latent spaces through entangling gates explicitly connecting the two, allowing it to leverage this information more effectively and achieve higher performance with fewer layers.

For the original ansatz, which employs all-to-all entanglement, the slower convergence can likely be attributed to its greater number of entangling gates. While these gates do not increase the number of trainable parameters, they create a high degree of interdependence between qubits, potentially leading to a more complex loss landscape. This interdependence may then make it more challenging for gradient descent to identify optimal parameter configurations efficiently. In contrast, the new ansatz introduces only sufficient entanglement to ensure efficient information transfer, avoiding the pitfalls of over-complexity and facilitating faster convergence with fewer layers.

For comparison of the new QAE architecture to CAEs, we turn to Tables 2 and 3. For Table 2, which considers the case of eight input features, the new single-layered ansatz contained 4 parameters, the shallow CAE 15, and for the deep CAE 183 parameters were used. Again, a performance separation is observed in favour of the new ansatz; however, for identifying the narrow graviton, all models performed similarly.

**Fig. 11:**
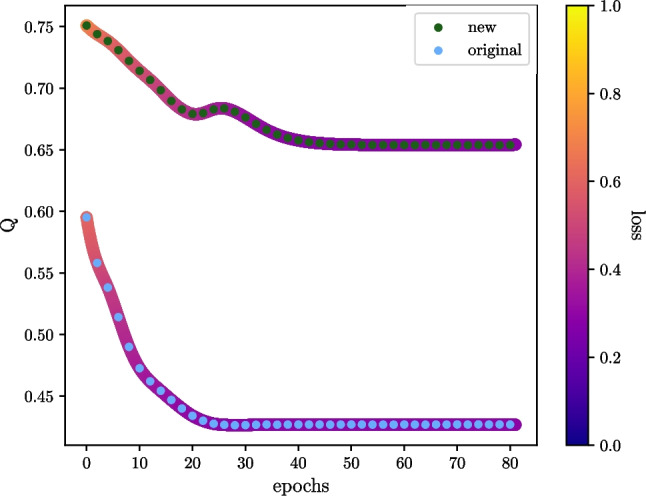
An illustration of how global entanglement varies during training for two types of ansatz sampled every two epochs averaged over five folds of training. The corresponding loss during training was recorded in the same manner as entanglement and has been included via a colour gradient; values for loss have been interpolated with a cubic spline. The validation set used for computing the above metrics was 5000 background samples. Data describing QCD background processes from the ‘Graviton and Scalar Boson’ dataset were used to construct this figure

Similarly, Table 3 (16 input features) shows a QAE with 24 parameters, a 42-parameter shallow CAE, and a more complex 810-parameter deep network. While CAEs narrowly outperform the QAE in identifying the narrow graviton (AUC, 0.997 vs 0.996), the QAE surpasses the best-performing CAE for both the scalar boson (AUC, 0.985 vs 0.978) and broad graviton (AUC, 0.731 vs 0.641) cases.

These results suggest that QAEs can achieve superior discriminating power compared to classical models using fewer or comparable numbers of parameters. This empirically demonstrates the potential for QAEs to achieve higher performance with lower computational complexity. We did also test overparamterised CAEs to see if they could exceed the performance of the best QAEs; however, they could only match the performance of the deep CAEs, and as such, we did not report them. A potential reason this form of model did not exceed the performance of the other classical models is that the dataset size was insufficient to fully leverage the advantages of over-parameterisation.

### Entanglement

We investigate the behaviour of global entanglement for a QAE during training and analyse how this entanglement evolves throughout the process, as illustrated in Fig. 11. For every two epochs, the global entanglement of the original and the new ansatz was calculated over five training folds using a validation set of 5000 samples. The observed trend is a decrease in global entanglement alongside decreasing loss function values, suggesting convergence to a state with less entanglement than is, on average, achieved by the ansatz.

**Fig. 12:**
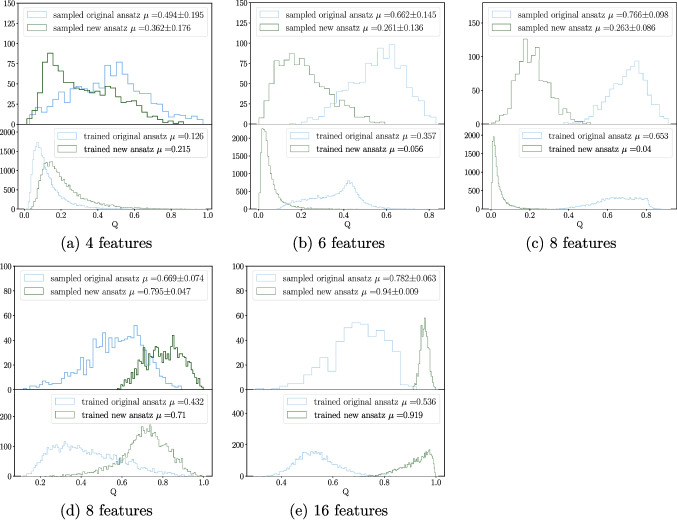
Histograms showing the amount of global entanglement generated by circuits using the Meyer-Wallach entanglement measure *Q*. Each subplot consists of two histograms; the lower histogram is the distribution of entanglement for a circuit over a set of background samples *x*. With a set of trained parameters $\theta _{trained}$, we can define the distribution via $Q(|\psi (x)|_{\theta _{trained}}\rangle )$. The upper histogram depicts a distribution given by $\mathbb {E}[Q(|\psi (\theta , x)\rangle$], where $\theta$ is sampled from the uniform distribution. This provides us a distribution on the expectation values of *Q* the circuit of interest could take. **a**, **b**, **c** Background samples are taken from the ‘heavy Higgs’ dataset, and the circuit sizes considered are four, six, and eight qubits going from left to right respectively. **d**, **e** Background samples are taken from the ‘scalar boson and graviton’ dataset, and the circuit sizes considered are four and eight qubits going from left to right respectively

To gain further insights, we present histograms of the global entanglement for circuits with randomly sampled parameters in Fig. 12. The top row shows circuits trained on background samples from the ‘heavy Higgs’ dataset, while the bottom row shows those trained on the background samples from the ‘scalar boson and graviton’ dataset. Each subplot consists of two histograms:

- **Lower histograms**: This represents the distribution of global entanglement over the dataset for a set of parameters found after training, denoted as $Q(|\psi (x)|_{\theta _{trained}}\rangle )$. The mean of this distribution is equivalent to Eq. 14.
- **Upper histograms**: This shows the distribution of global entanglement for the dataset across multiple randomly sampled parameter sets, represented by $\frac{1}{N}\sum _i^NQ(|\psi (x_i,\theta )\rangle )$. The mean of this distribution is equivalent to Eq. 15.

Across all plots in Fig. 12, a consistent trend emerges. The final, optimised parameters consistently generate a global entanglement with a lower expected value compared to the bulk of the distribution arising from uniformly sampled parameters. In the cases shown for the new ansatz, all but the first plot, which considers four input features from the ‘heavy Higgs’ dataset, show $\mathbb {E}[Q(|\psi (x)|_{\theta _{trained}}\rangle )]$ to be over $1\sigma$ from $\mathbb {E}[\frac{1}{N}\sum _i^NQ(|\psi (x_i,\theta )\rangle )]$. This suggests that the QAE exhibits a preference for lower entanglement states. However, it is important to note that these states do not represent the minimum entanglement achievable by these circuits. This indicates a preference for a moderate degree of entanglement, though not an excessive amount.

One potential explanation for this preference is that high entanglement levels might lead to information scrambling, making lower entanglement states more favourable. Previous work explored this idea, showing that too much entanglement can render quantum states useless for quantum computation (Gross et al. 2009; Ortiz Marrero et al. 2021).

**Fig. 13:**
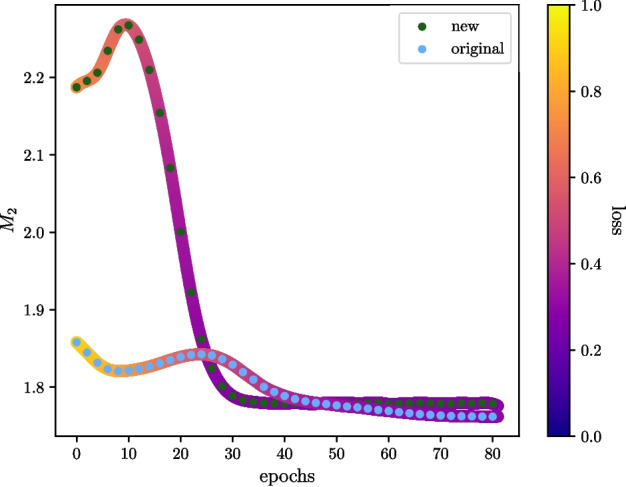
An illustration of how magic varies during training for two types of ansatz sampled every two epochs averaged over five folds of training. The corresponding loss during training was recorded in the same manner as magic and has been included via a colour gradient, and values for loss have been interpolated with a cubic spline. The validation set used for computing the above metrics was 1000 background samples. Data describing QCD background processes from the ‘Graviton and Scalar Boson’ dataset were used to construct this figure

Furthermore, focusing on the top row of Fig. 12, we observe that the entanglement generated by the new ansatz remains localised with increasing problem size. In contrast, the original ansatz exhibits a flattening in its distribution with increasing problem size, coinciding with its observed decrease in performance.

**Fig. 14:**
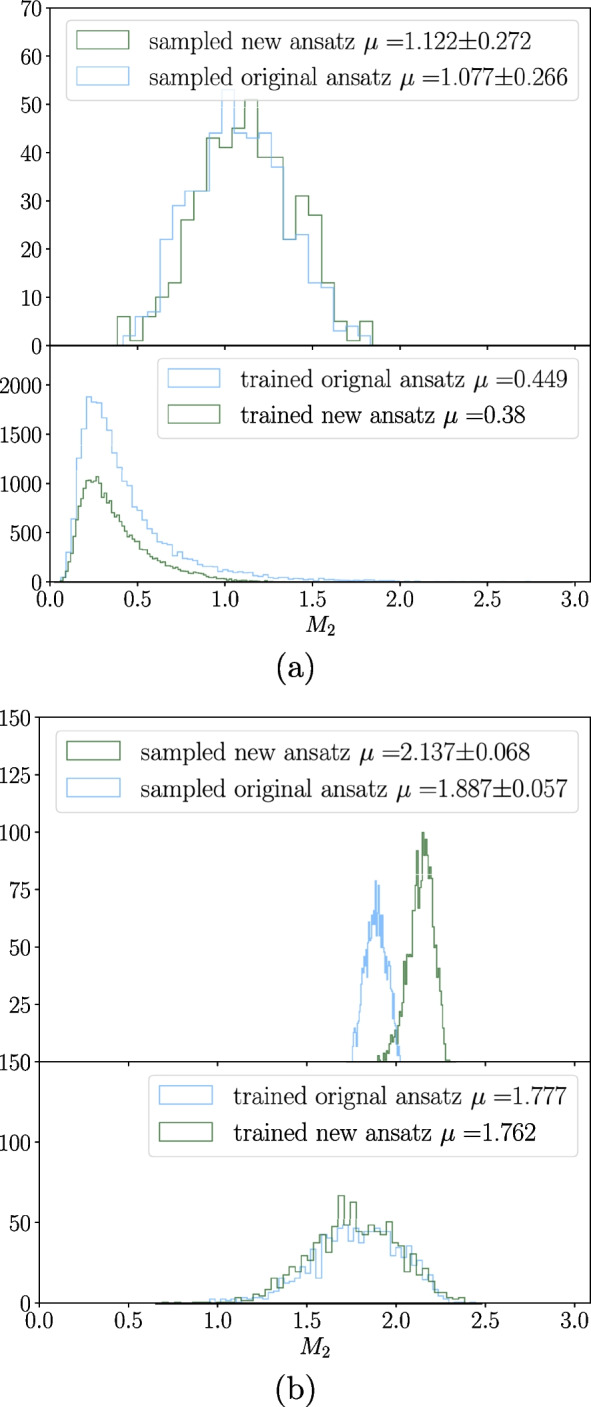
A collection of histograms depicting the amount of magic generated by circuits using the stabiliser Rényi entropy $M_2$. Each subplot consists of two histograms; the lower histogram is the distribution of magic for a circuit over a set of background samples *x*. With a set of trained parameters $\theta _{trained}$, we can define the distribution via $M_2(|\psi (x)|_{\theta _{trained}}\rangle )$. The upper histogram depicts a distribution given by $\mathbb {E}[M_2(|\psi (\theta , x)\rangle$], where $\theta$ is sampled from the uniform distribution. This provides us a distribution on the expectation values of $M_2$ the circuit of interest could take. **a** Background samples are taken from the ‘heavy Higgs’ dataset, for a circuit size of four qubits. **b** Background samples are taken from the ‘scalar boson and graviton’ dataset for a circuit size of four qubits

A final observation is that the entanglement decreases for the new ansatz as the problem size increases when considering the ‘heavy Higgs’ dataset in the top row of Fig. 12. Meanwhile, for the new ansatz, entanglement increases when considering the ‘scalar boson and graviton’ dataset in the bottom row of Fig. 12. The exact reasons for these trends may depend on factors such as entanglement in the feature map or the data itself. No entanglement was present in the feature map for the ‘heavy Higgs’ dataset, but for the ‘scalar boson and graviton’ dataset, there was. This could be a topic for further exploration since entanglement growth is necessary in order to avoid dequantisation (Eisert 2013). Moreover, recent findings show that entanglement of the input data can have an impact on the trainability of the specific ansatz chosen (Leone et al. 2024).

### Magic

Following our analysis of entanglement, we adopt a similar approach to investigate the behaviour of magic during training. Figure 13 shows the magic generated by the original and new ansatz, recorded every two epochs over five training folds with a validation set of 1000. Similar to the observed trend for global entanglement, we see a decrease in magic alongside decreasing loss function values, suggesting convergence towards lower magic states.

Analogous to the entanglement analysis, Fig. 14 presents histograms of magic with uniformly randomly sampled parameters. The *x*-axis limits on the histograms are purposely set to be the minimum and maximum attainable magic for the system being considered. The subplots depict the following:

- **Lower histograms**: This represents the distribution of magic over the dataset for a set of parameters found after training, denoted as $M_2(|\psi (x)|_{\theta _{trained}}\rangle )$. The mean of this distribution is equivalent to Eq. 17.
- **Upper histograms**: This depicts the distribution of magic for the dataset across multiple randomly sampled parameter sets, represented by $\frac{1}{N}\sum _i^NM_2(|\psi (x_i,\theta )\rangle )$. The mean of this distribution is equivalent to Eq. 18.

As evident in Fig. 14, the magic generated by circuits with optimised parameters consistently falls to the left of the mean compared to randomly sampled circuits. This effect is more pronounced for the ‘heavy Higgs’ dataset than the ‘scalar boson and gravitons’ dataset. It is interesting that, in fact, the most valuable states are those with less magic. Since this is one of the first studies into entanglement and magic simultaneously for PQCs, further research is required to explore the interplay between these quantities and the usefulness of states with certain levels of each quantity.

## Conclusion and outlook

In conclusion, we investigated and improved upon two existing studies in anomaly detection in high-energy physics by proposing a novel QAE ansatz, considering up to 16 features. The new QAE ansatz demonstrated a performance advantage in both datasets compared to previously considered ansatz. This may suggest that the ansatz is applicable across a variety of datasets. This study addresses larger, more relevant problem sizes in the context of particle physics. However, future studies using even larger problem sizes will be key to verifying that the trend for QML models matching or beating their classical counterparts using fewer parameters continues to hold.

Our study highlights the importance of designing ansatz that respects the problem’s inherent structure. The newly developed QAE, which incorporates this principle, outperformed previously considered ansatz despite all three sharing the same single-qubit rotation gates. This finding suggests that the specific entangling structure can significantly impact performance. In particular, it allows for scaling to more qubits, which is not observed for previously used models. Future work could investigate whether this ansatz design offers advantages for datasets beyond LHC physics, as its data-agnostic construction suggests broader applicability.

We observed a clear separation in performance between the QAE and CAEs, with the QAE often requiring significantly fewer parameters to achieve superior performance in both datasets. Additionally, we explored the behaviour of global entanglement and magic generated by quantum circuits during training, demonstrating a convergence towards values smaller than the expectation value for the circuit family in both metrics.

Further studies could investigate the scaling of entanglement and magic with increasing problem size to determine if optimised circuits possess sufficient levels of the two to resist dequantisation methods such as tensor networks (Shin et al. 2024; Pan et al. 2022; Cerezo et al. 2023). Moreover, we believe it is important to understand the origin behind this observed preference for lower entanglement and magic during optimisation. This could involve analysing the relationships that entanglement and magic may have with the feature map used.

## Figures and Tables

**Algorithm 1:**
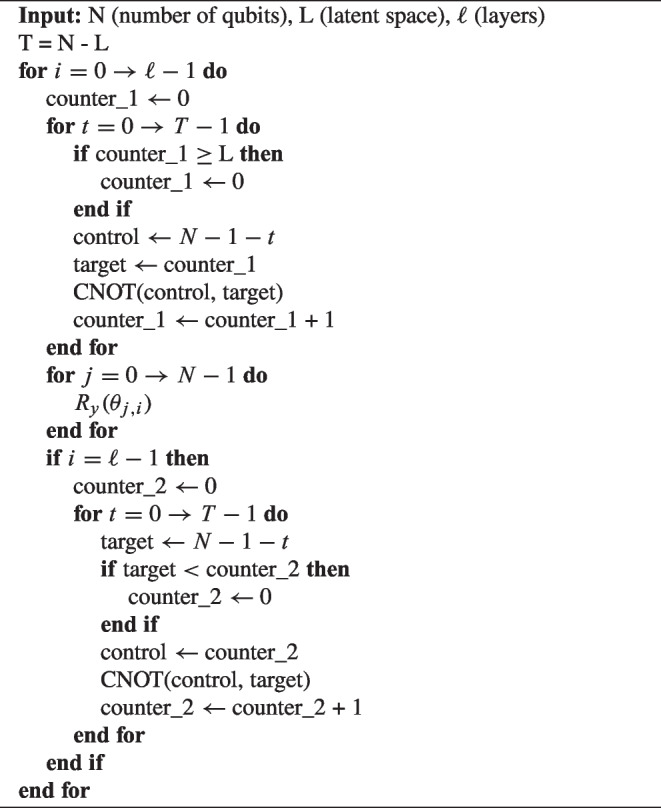
New ansatz pseudo-code.

## Data Availability

The datasets analysed are publicly available and have been referenced in the manuscript. Additional code used to analyse these datasets during the current study are available from the corresponding author on reasonable request.

## Appendix A: New ansatz pseudocode

The accompanying pseudocode for the newly proposed ansatz for the QAE.

## Appendix B: Classical and quantum autoencoder details

The tables presented here detail the hyperparameters of the QAEs and CAEs used in the above studies. Not listed in the tables for the CAEs are the activation functions used; these were leaky-relu functions for all hidden layers bar those connecting to the latent space which made use of the linear activation function. A learning rate of 0.001 along with the Adam optimiser was used for the CAEs and QAEs (Tables 4, 5, 6 and 7).

**Table 4:** Details of the classical autoencoders used for identifying the heavy Higgs signal depicted in Table 1

Input size	Batch size	Hidden layers	Epochs	Sparsity
4 (shallow)	500	[4,3,1]	500	0.400
4 (deep)	500	[4,4,3,2,1]	500	None
6 (shallow)	500	[3,3,2]	500	0.305
6 (deep)	500	[8, 8, 8, 5, 4]	500	None
8 (shallow)	500	[5, 4]	500	0.553
8 (deep)	500	[32, 32, 8, 4]	500	0.750

**Table 5:** Details of the quantum autoencoders used for identifying the heavy Higgs depicted in Table 1

Input size	Batch size	Layers	Epochs	Latent space
4	50	1	500	1
6	50	1	500	3
8	50	1	500	4

**Table 6:** Details of the quantum autoencoders used for identifying a scalar boson and gravitons depicted in Tables 2 and 3

Input size	Batch size	Layers	Epochs	Latent space
8	50	1	500	2
16	50	3	500	4

**Table 7:** Details of the classical autoencoder used for identifying a scalar boson and gravitons depicted in Tables 2 and 3

Input size	Batch size	Hidden layers	Epochs	Sparsity
8 (shallow)	500	[1]	500	0.731
8 (deep)	500	[5, 4, 3, 1]	500	None
16 (shallow)	50	[2]	500	0.652
16 (deep)	500	[8, 6, 5, 5, 1]	500	None

## Appendix C: Grid search

The grid search parameters used to determine the most performant models (Tables 8, and 9).

**Table 8:** Details of the grid search used to identify classical autoencoders

Hyperparameter	Values
Batch sizes	[50,500,1000]
Hidden layers	Range [1,5]
Neurons	Range [1,32]
Latent space	Range [1,4]
Prune	Range [0,1]

**Table 9:** Details of the grid search used to identify quantum autoencoders

Hyperparameter	Values
Batch sizes	[50,500,1000]
Hidden layers	Range [1,6]
Latent space	Range [1,5]

## Footnotes

1 Dataset available at: https://gitlab.com/vishalng/qae_ hep.

2 Dataset available at: https://doi.org/10.5281/zenodo.7696203
